# Ion Channel Regulation by Sex Steroid Hormones and Vitamin D in Cancer: A Potential Opportunity for Cancer Diagnosis and Therapy

**DOI:** 10.3389/fphar.2020.00152

**Published:** 2020-02-28

**Authors:** Iván Restrepo-Angulo, Cecilia Bañuelos, Javier Camacho

**Affiliations:** ^1^ Department of Pharmacology, Centro de Investigación y de Estudios Avanzados del Instituto Politécnico Nacional, Mexico City, Mexico; ^2^ Transdisciplinary Program on Science, Technology and Society, Centro de Investigación y de Estudios Avanzados del Instituto Politécnico Nacional, Mexico City, Mexico

**Keywords:** steroid hormones, ion channels, cancer, cancer therapy, tumor markers

## Abstract

Many ion channels are involved in tumor development, promoting cancer cell proliferation, migration, invasion, and survival. Accordingly, some of them have been suggested as tumor markers and novel targets for cancer therapy. Some sex steroid hormones (SSH), including estrogens and androgens, favor cancer progression. Meanwhile, other steroid hormones like vitamin D may have anticancer properties. SSH and vitamin D modulate the expression of a number of ion channels in cancer cells from hormone-sensitive tissues, including breast, ovary, prostate, and cervix. Moreover, rapid effects of SSH may be mediated by their direct action on membrane ion channels. Here, we reviewed the SSH and vitamin D regulation of ion channels involved in cancer, and analyzed the potential molecular pathways implicated. In addition, we described the potential clinical use of ion channels in cancer diagnosis and therapy, taking advantage of their regulation by SSH and vitamin D. Since SSH are considered risk factors for different types of cancer, and ion channels play important roles in tumor progression, the regulation of ion channels by SSH and vitamin D may represent a potential opportunity for early cancer diagnosis and therapeutic approaches in SSH and vitamin D sensitive tumors.

## Introduction

Sex steroid hormones (SSH) have been considered as a risk factor to develop a variety of human cancers, including those from breast, endometrium, ovary, cervix, colon, and prostate (Maxwell et al., 2009). These hormones act as tumor promoters by inducing proliferation and preventing apoptosis of mutated cells, thus, the possibility of additional genetic and/or epigenetic changes is enhanced (Irigaray and Belpomme, 2010). From a classical point of view, SSH interact with their intracellular receptors, which bind to specific regions of genes involved in cell proliferation and/or differentiation (Bohra and Bhateja, 2015).

Cumulative evidence has shown that ion channels have a role in carcinogenesis by participating in pathways that lead cancer cells to proliferate, survive, migrate, and invade (Kunzelmann, 2005; Prevarskaya et al., 2018). Many ion channels favor tumor progression in hormone-sensitive tissues, hence, the immediate arising question is whether SSH may regulate the expression or activity of ion channels involved in cancer. As the reader will find in this review, SSH exert the transcriptional control of some genes encoding for ion channels. Moreover, SSH not only regulate the activity of ion channels through the canonical transcriptional pathway, besides, they can also bind to channel domains and alter their gating properties, resulting in rapid cellular responses.

This review is intended to compile and show evidence about how ion channels are involved in the carcinogenesis process primarily induced by SSH, namely estrogens and androgens. We also include vitamin D in this review because— although it is not an SSH—it is a steroid hormone (SH) displaying anti-proliferative effects, and regulates some ion channels involved in cancer in hormone-sensitive-tissues. Altogether, ion channels have tremendous potential for translational research as biomarkers for cancer diagnosis and prognosis, as well as for improving the treatment of SH-associated tumors.

## Steroid Hormones: An Overview

SH regulate a wide variety of developmental and physiological processes from fetal life to adulthood. These hormones are derived from cholesterol; therefore, they have a closely related structure based on the cyclopentanophenantrene (Miller and Auchus, 2011). They are synthesized and secreted by the adrenal cortex, ovaries, testes, and during pregnancy, by the placenta. Considering their function, steroids can be classified into two major classes: A) *corticosteroids*, which are in turn divided into glucocorticoids, like cortisol and cortisone, which regulate glucose metabolism, and mineralocorticoids, such as aldosterone, that helps to regulate the concentration of sodium and potassium ions in blood; and B) *sex steroids*, which are responsible for the maturity of the reproductive structures, among other functions. Three SSH are known: *estrogens (estradiol),* contributing to the development and maintenance of female secondary sex characteristics; *androgens (testosterone),* that participate in the development and maintenance of male secondary sex characteristics; and *progestagens (progesterone)*, involved in regulating the cyclic changes experienced by the endometrium (Chatuphonprasert et al., 2018). Other SH also comprise *neurosteroids* (those synthesized in the brain, like *allopregnolone*) and *vitamin D,* which is an open-ring steroid, considered as a pro-hormone, because it needs to be transformed in the kidney into its active metabolite, namely 1α,25 dihydroxivitamin D (1,25 VD), also known as calcitriol (Norman, 2008). Throughout the text, SSH will refer to estradiol, progesterone, and testosterone, while SH will include also other hormones like vitamin D.

SH act through receptors located in the cell cytoplasm to control the expression of their target genes. In this transcriptional pathway, the hormone binds to its receptor, inducing a receptor-conformational change. Subsequently, the hormone-receptor complex translocates to the nucleus, where it interacts with specific sequences on the DNA, ultimately resulting in either the up- or down-regulation of the target gene. Transcriptional control of gene expression may take from some hours to a number of days. Examples of these types of receptors are the estrogen receptors alpha and beta (ERα and Erβ), the androgen receptor (AR), and the vitamin D receptor (VDR) (Wilkenfeld et al., 2018). Along with the transcriptional pathway, SH induce rapid, non-transcriptional effects, including blood vessels dilation or tracheal relaxation. It has been proposed that such non-transcriptional actions are produced by hormone binding to either G-protein-coupled membrane receptors or the classical intracellular receptor types embedded into the cell membrane. Examples of these types of receptors are the GPR30 receptor for estrogens (Meyer et al., 2011; Reslan and Khalil, 2012; Wang et al., 2014; Chen et al., 2019), variants of the ERα (Pietras and Márquez-Garbán, 2007; Adlanmerini et al., 2014; Arnal et al., 2017), or the calcium ion channel CatSper, which has a binding domain for prostaglandins and mediates progesterone-induced Ca^2+^ influx in human sperm (Strünker et al., 2011).

Many ion channels regulated by SH in normal cells and tissues have not been yet associated with cancer. However, recognizing such regulation in healthy conditions may provide hints for its potential inclusion in cancer research, as well as to improve our understanding of the participation of SH in different malignancies. Thus, we will review first some examples of the effects of these hormones on ion channels in normal conditions.

## Steroid Hormones Regulate the Expression and/or Activity of Ion Channels in Normal Physiological Events

There is a myriad of genes transcriptionally regulated by SH, including a number of ion channels (Kim et al., 2006; Lee and Jeung, 2007; Fraser et al., 2014). Something really fascinating is that SH may also regulate the activity of ion channels by acting on its gating properties, either by inducing the expression of ion channel-interacting proteins, or by direct binding to channels (Majewska et al., 1986; Valverde et al., 1999; Herd et al., 2007; Strünker et al., 2011; Kow and Pfaff, 2016; Morales-Lázaro et al., 2019). The effect of SH on ion channels seems to be a relevant mechanism by which steroids meet many of their physiological functions in both, the cardiovascular and nervous systems, as well as in non-excitable tissues. Next, some examples of these effects in non-cancer cells are discussed.

### Cardiovascular System

In general, women at ages before the beginning of menopause are less vulnerable than men to present cardiovascular diseases (Pabbidi et al., 2018). K_Ca_1.1 channels are key elements in the modulation of the vascular smooth muscle tone. One of the most interesting findings associating SSH and ion channels was reported by the end of the last millennium (Valverde et al., 1999), describing that 17 β-estradiol directly binds to the β-regulatory subunit of the KCa1.1 channel, leading to channel activation. More recently, Granados et al. (2019), reported that the estradiol-binding site was located in residues present in the second transmembrane domain of the channel β1 subunit, and that the increase in the open probability of the channel induced by 17 β-estradiol (10 μM) was associated to the voltage-sensor stabilization. These observations suggest a rapid and direct mechanism by which estrogens regulate vascular smooth muscle relaxation, protecting women from cardiovascular diseases in some hormonal conditions. Estrogens also increase the expression of the β1 subunit of this channel in the uterine arterial smooth muscle cells during pregnancy. This increase, in turn, enhances the calcium-dependent opening of channels, resulting in artery dilation (Sakamoto and Kurokawa, 2019).

In cardiomyocytes, progesterone enhances the cardiac slow delayed rectifier potassium current (Iks), and suppresses the L-type calcium current through a non-transcriptional pathway that involves sequential activation of c-Src, PI3-kinase, Akt, eNOS, and nitric oxide (NO) (Kurokawa et al., 2016). Likewise, testosterone promotes Iks current and blocks L-type calcium current in guinea pig cardiac muscle cells (Sakamoto and Kurokawa, 2019). These currents shape the repolarization phase of the cardiac action potential. Therefore, by acting on the L-type (Cav1.x) Ca^+2^ channels and the delayed rectifier K^+^ channels, SSH may be partly responsible for the differences in the cardiac electrical activity observed between women and men. However, the role of estradiol in modulating the electrical activity of the heart is not completely understood. Chronic administration of estradiol down-regulates the mRNA levels of the voltage-gated potassium channels Kv1.5 and Kv7.1 in the ventricle of ovarectomized rabbits (Drici et al., 1996). Additionally, estradiol inhibits the rapid delayed rectifier potassium current (Ikr), and increases the duration of the cardiac action potential, as well as the QT interval at physiological concentrations. At higher concentrations, estradiol inhibits Ikr and enhances Iks currents by non-transcriptional pathways (Kurokawa et al., 2016). Moreover, Anneken et al. (2016), showed that estradiol enhances the interaction between chaperone proteins and the subunits of the inward rectifying potassium channel Kv11.1 (KCNH2, ether-à-go-go related gene, ERG), leading to an increased trafficking of the channel towards the cell membrane. This channel is a key element for generating Ikr currents. Estradiol interacts with Kv11.1 channels through their phenylalanine residue 656 (Phe^656^) at physiological concentrations, and enhances the effect of the selective Kv11.1 channel blocker E-4031. This effect was ablated in aromatase-null mice (Kurokawa et al., 2015), which may explain why some drugs induce long QT in women (Darpo et al., 2014). Some drugs used in breast cancer chemotherapy target either ERα or estrogen synthesis. This hormone-therapy may have either vascular side-effects or even increase the risk of endometrial cancer (for instance in the case of tamoxifen) (Heery et al., 2018). Thus, the effect of estradiol on vascular and cancer-associated ion channels like Kv11.1 or K_Ca_1.1 should be taken into consideration when breast cancer patients receive hormone-therapy.

### Neuro-Protection and Neuronal Firing

Increasing evidence suggests that ion channels participate in the protective role of steroids in the central nervous system. For instance, 17-β estradiol significantly prevents glutamate-induced apoptosis in ventro-spinal motoneurons, and in the neuroblastoma hybrid cell line VSC4.1. Estradiol exerts this effect by decreasing intracellular concentrations of Ca^+2^ and caspase activity. Interestingly, the Cav1.x calcium channel blocker nifedipine also achieves a neuro-protective level, seemingly to that observed in cells treated with 17-β estradiol. In addition, 17-β estradiol also inhibited the pro-apoptotic effect induced by the Ca^+2^ channel agonist FPL-64176. These results suggest that 17-β estradiol might prevent apoptosis in neurons by reducing the calcium influx through Cav1.x calcium channels (Sribnick et al., 2009). Sánchez et al. (2014), showed that 17-β estradiol significantly reduced calcium currents flowing through Cav1.x channels in rat cultured cortical neurons, which were also inhibited by the neuro-protective insulin-like growth factor 1 (IGF-1). Conversely, another report showed that 17-β estradiol at picomolar concentrations binds to the dihydropyridine site in the Cav1.x calcium channels, enhancing calcium currents (Vega-Vela et al., 2017). Therefore, the molecular mechanisms involved in Cav1.x channel regulation by estradiol require further investigation. Progesterone also has a protective effect on striatal neurons in a glutamate-induced apoptosis model. Interestingly, progesterone exerts its anti-apoptotic effect by blocking the Cav1.x calcium channel. Such blockade occurs at higher concentrations than those required to activate the progestin receptor, suggesting that the progesterone receptor is likely not necessary for the observed effect of progesterone. Moreover, dose-response experiments showed that at 100 μM, progesterone completely abolished whole-cell calcium currents, indicating that this hormone may affect other types of calcium channels (Luoma et al., 2012).

Estradiol is also involved in modulating the excitability of neurons in different brain nucleus. Zhang et al. (2015) showed that the pore-forming subunit of the voltage gated sodium channel Nav1.1α, and its auxiliary β2-subunit, were two to three fold up-regulated by estradiol (subcutaneous injection of 0.25 μg 17-estradiol benzoate) in neurons of the anteroventral/periventricular preoptic nucleus. This channel plays an important role in maintaining persistent sodium currents, providing these cells with a spontaneous and repetitive burst firing. This firing is required for the high-frequency-stimulated release of kisspeptin, which in turn promotes the activity of neurons expressing the gonadotropin-releasing hormone. Since some neurotransmitters favor the proliferation, migration and invasion of cancer cells (Jiang et al., 2020), the regulation of neuronal firing by SSH *via* ion channels may provide hints of novel targets for cancer therapy.

### Non-Excitable Tissues

A number of interesting reports about the regulation of the activity and expression of ion channels by steroids hormones in non-excitable tissues have been published. Aldosterone induces the expression of the epithelial sodium channel (ENaC) in the colon and kidney. This channel has a pivotal role in sodium re-absorption, which influences the volume of body fluids and blood pressure (Snyder, 2002). Aldosterone also regulates ENaC channels through non-transcriptional mechanisms, by promoting channel trafficking from intracellular compartments to the plasma membrane (Palmer and Frindt, 2016). Estrogens modulate the activity of ENaC channels in the lung epithelium. The open probability of the ENaC channels and its number increased in the rat-alveolar cell line L2 after estradiol treatment. Furthermore, a comparison of lung homogenates from female rats in pro-estrous and di-estrous phases revealed that the highest number of ENaC channels expressed in the cell membrane parallels the highest level of circulating estradiol (Matalon et al., 2015).

Head et al. (1999) observed that progesterone rapidly activates an inward current and increases the intracellular calcium concentration in thymulin-secreting epithelial cells from rat thymus. Because of the rapid response to progesterone, such effect had likely been mediated by a non-transcriptional pathway requiring the activation of a second messenger. Progesterone also enhances the activity of the sperm-specific calcium channel CatSper, by binding the enzyme ABHD2, which promotes the removal of the CatSper inhibitor 2-arachinodoylglycerol (2-AG) (Mannowetz et al., 2017).

Pregnancy is a typical example of a normal physiological hyper-estrogenic condition, thus, it is expected that channels regulated by SSH participate during this process. Accordingly, Kv10.1 (KCNH1, ether-à-go-go-1, EAG1) potassium channel mRNA levels were up-regulated by estradiol in primary cultures from human placental trophoblasts, and human vascular endothelial cells (HUVEC cell line) (Díaz et al., 2009). This effect was prevented by an ERα antagonist. Moreover, potassium currents resembling Kv10.1 channel activity were enhanced by estradiol treatment in syncitium-trophoblasts. Kv10.1 channels are also up-regulated by estradiol in the cervix during pregnancy. While Kv10.1 protein expression in cervical cells was observed in only 26% of non-pregnant women, it was found in 100% of pregnant patients, independently of the trimester of pregnancy or human papillomavirus (HPV) infection (Ramírez et al., 2013). Nevertheless, the precise role of Kv10.1 channels in the placental and cervical tissue remains to be elucidated. The hormonal regulation of ion channels in normal non-cancerous tissues may provide clues to find novel early tumor markers. Following, we will review the regulation of ion channels by SSH and calcitriol in diverse cancer models.

## Sex Steroid Hormones and Vitamin D Control The Expression and Activity of Ion Channels In Cancer

The regulation of ion channels by estradiol, progesterone, androgens, and calcitriol in different types of cancer has been reported, mainly at the gene and protein expression level, as well as channel activity.

### Estrogenic Regulation of Ion Channels

#### Potassium Channels

SSH modulate the expression and activity of ion channels in cancerous epithelial cells. Díaz et al. (2009) showed that 17-β estradiol, at very low concentrations (10^-10^ M), significantly up-regulated the expression of Kv10.1 channels in the cervical cancer cell line HeLa transfected with ERα. Interestingly, no significative increase in Kv10.1 mRNA levels was observed in HeLa cells lacking ERα. Carlson et al. (2013) pointed out that the effect of 17-β estradiol on Kv10.1 occurs at transcriptional level, since no changes in its biophysical properties were seen when channels were exposed to estradiol concentrations up to 50 µM. Likewise, Kv10.1 mRNA and protein levels were increased in a dose-dependent manner by progesterone (10^-8^ M, 48 h) in HeLa cells bearing the progesterone receptor (Ramírez et al., 2013). The expression of this channel and its regulation by estrogens has also been studied in a transgenic mouse model for cervical cancer development (Ramírez et al., 2018). Transgenic mice expressing the E7 oncogene of HPV and treated with estradiol (0.05 mg pellets subcutaneously inserted), for 3 or 6 months, developed cervical dysplasia and cervical cancer, respectively. Moreover, Kv10.1 mRNA and protein expression were significantly increased by estradiol. The highest expression was found in animals with cervical cancer. Since SSH are considered potential co-factors for cervical cancer development, the regulation of Kv10.1 channels by estradiol and progesterone provides a supplementary mechanism by which these hormones may contribute to cervical cancer progression.

KCa1.1 potassium channels are very interesting proteins because they are overexpressed in hormone-sensitive tumors, and have been involved in different functions relevant in cancer, including cell proliferation and migration (Ge et al., 2014). In addition to their overexpression in gliomas (Liu et al., 2002), their expression is enhanced in tumors influenced by hormones, including breast, ovarian, prostate, and cervical cancer (Bloch et al., 2007; Oeggerli et al., 2012; Ramírez et al., 2018). Extracellular perfusion of 17-β estradiol increased the current flowing through the Maxi-K channels in MCF-7 epithelial breast cancer cells. This effect was not abrogated by treating the cells with the ERα antagonist ICI-182, and it was not necessary for 17-β estradiol to diffuse into the cell to exert its effect on channels (Coiret et al., 2005). The authors concluded that the opening of KCa1.1 channels by 17-β estradiol may represent an alternative pathway by which this hormone induces cell proliferation of epithelial breast cancer cells. In accordance, the inhibition of channel expression or activity with specific antisense probes or blockers reduced the hormone-induced activation of the channel, as well as cell growth, in breast and prostate cancer cells (Bloch et al., 2007; Oeggerli et al., 2012). This channel was also studied in the HPV-transgenic mice above mentioned. Similarly to Kv10.1 channels, KCa1.1 mRNA and protein expression was significantly increased by estradiol, and the highest expression was found in animals with cervical cancer (Ramírez et al., 2018). Interestingly, multiple estrogen-responsive sequences have been found in the gene promoter of KCa1.1 channels (Kundu et al., 2007). 17-β estradiol also regulates the expression of KCa1.1 channels in endometrial cancer cells. Wang et al. (2018) observed that Ishikawa cells (a human ERα positive endometrial adenocarcinoma cell line) treated with 17-β estradiol (1 nM) presented an increased expression of the KCa1.1 channel protein, as well as phosphorylated ERK (p-ERK) and MEK (p-MEK). Remarkably, by knocking-down the expression of the KCa1.1 channel, the protein levels of p-ERK and p-MEK were also down-regulated. KCa1.1 knockdown also resulted in decreased proliferation, migration, and invasion of Ishikawa cells. These results indicate that KCa1.1 channels could be essential molecules mediating the effects of 17-β estradiol on endometrial cancer Ishikawa cells.

#### Calcium Channels

Different types of calcium channels have a crucial role in the carcinogenesis process induced by 17-β estradiol. Motiani et al. (2010) showed that ERα-positive breast cancer cell lines (MCF-7, BT474, ZR751, T47D, and HCC1500) display higher protein expression of the store-operated calcium channel Orai3, compared to ERα-negative breast cancer cells. The store-operated calcium entry in ERα-positive breast cancer cells was mediated by Orai3 channels, then, it was proposed that 17-β estradiol induces the expression of these channels in ER positive breast cancer cells. Later, the same group (Motiani et al., 2013) reported that ERα knockdown decreased Orai3 mRNA and protein expression. Additionally, it also reduced store-operated calcium entry, inhibiting the phosphorylation of ERK 1/2 and FAK, and preventing the activation of the transcription factor NFAT1. Orai3 knockdown also decreased the anchorage-independent growth and matrigel invasion of MCF-7 cells, as well as tumorigenesis in mice treated with estradiol (0.72 mg/pellet subcutaneously implanted) (Motiani et al., 2013).

The melastatin transient receptor potential 8 (TRPM8) calcium channel is highly expressed in MCF-7 cells. Interestingly, this channel is significantly down-regulated after culturing cells in estrogen-free medium for 48 to 72 h. However, TRPM8 expression levels can be restored by adding 17-β estradiol (10 nM). ERα knockdown also depressed the levels of TRPM8 mRNA. Furthermore, breast cancer clinical samples display increased expression of TRPM8 channels associated with ERα tumor status (Chodon et al., 2010).

Hao et al. (2015) reported that 17-β estradiol regulates the expression of the subunits Cav1.3—Cav1.4 of the L-type voltage-gated calcium channels. Estradiol increased the expression of Cav1.3 in endometrial cancer cells in a dose- and time-dependent manner. Interestingly, the cell-impermeable protein-conjugated 17-β estradiol (100 nM) also elicited a rapid increase of Cav1.3 expression, which peaked at 30 min. The expression of the Cav1.3 channel was inhibited by pertussin toxin (200 ng/ml), which is a well-known inhibitor of G-coupled protein receptors. A small interfering RNA targeting the GPR30, also inhibited the effect of estradiol-induced up-regulation of the Cav1.3 subunit, and significantly reduced the phosphorylation of ERK ½ and CREB. Moreover, Cav1.3 knockdown markedly impaired estrogen-stimulated calcium influx, cell proliferation, and migration of endometrial cancer cells. Altogether, these data suggest that Cav1.3 is an important component of the molecular setting activated during the carcinogenesis process induced by 17-β estradiol in endometrial cells (Hao et al., 2015).

TRPV6 channels are overexpressed in mammary adenocarcinoma tissue, and play a key role in calcium homeostasis (Zhuang et al., 2002). In accordance, their specific knockdown with siRNA decreased calcium influx by 44% compared to control siRNA. Moreover, cells with TRPV6- targeted knockdown showed less viable cells at 24 and 48 h (35 and 40%, respectively), as well as an increased number of cells in G1 phase at 24 h (Peters et al., 2012). Bolanz et al. (2008) reported that 17-β estradiol (10 nM) up-regulated the expression of TRPV6 in T-47D breast cancer cells, in a time-dependent manner from 24 h of exposure. After 72 h of treatment, TRPV6 mRNA levels increased 69% compared to the basal level. Altogether, these results strongly suggest that TRPV6 channels facilitate the calcium influx required for breast cancer cells to proliferate and survive, and that these channels are part of the molecular mechanism of the 17-β estradiol-induced proliferation in breast cancer cells.

#### Sodium Channels

Voltage-gated sodium channels are up-regulated in breast cancer. It has been proposed that they potentiate cell motility, endocytosis, and invasion (Mao et al., 2019). These channels are overexpressed in different types of cancers including hormone-sensitive tumors like those from prostate, cervix, and breast. Channel activity and/or expression is even more enhanced in metastatic cells, and channel inhibitors decrease their invasive properties (Diaz et al., 2007; Mao et al., 2019). In the case of breast cancer, the expression of the neonatal spliced form of the Nav1.5 channel has been strongly associated with high metastatic potential *in vitro*, and breast cancer progression *in vivo* (Fraser et al., 2005). Interestingly, there is an inverse correlation between the expression of Nav1.5 and the expression of ERα in breast cancer cells. The metastatic breast cancer cell line MDA- MB-231 lacks ERα and expresses Nav1.5. Conversely, the weak non-metastatic cell line MCF-7 cells expresses ERα, but shows very low expression levels of Nav1.5 (Fraser et al., 2014; Mohammed et al., 2016). Accordingly, MDA-MB-231 cells stably transfected with the ERα, show a significant decrease in the expression and activity of Nav1.5 channels. The channel expression and function were restored after treating cells with the ERα antagonist ICI- 182 780 (1 μM) for more than 48 h (Fraser et al., 2014). In this case, the GPR30 acted in opposition to ERα, although its action seems to involve a non-transcriptional pathway. Extracellular application of 17-β estradiol (10 nM) to MDA-MB-231 cells for a short time (10 s), increased Nav1.5 currents *via* GPR30-dependent activation of PKA, leading to a reduction of cell adhesiveness (Fraser et al., 2010). These results suggest that estradiol might have either, pro- or anti-metastatic effects, depending on the estrogen receptor found in breast cancer cells. Further investigation is needed to elucidate how these pathways may function in patients.

#### Chloride Channels

Chloride channels participate in different processes of neoplastic transformation, including cell proliferation, migration, invasion, and metastasis (Peretti et al., 2015). Evidence provided by Yang et al. (2018) suggests that the expression of the voltage-gated chloride channel CLC-3 is regulated by 17-β estradiol in breast cancer cells. MCF-7 cells treated with 17-β estradiol showed increased chloride currents that were blocked by 5-nitro-2-(3-phenylpropil-amino) benzoic acid (NPPB), and 4-4´ diisothiocyanatostilbene-2, 2´ disulfonic acid (DIDS). Moreover, data point out to a transcriptional control *via* ERα, since CLC-3 currents were detected in MDA-MB-231 transfected with the ERS1 gene coding for ERα. CLC-3 currents disappeared after treating MCF-7 cells with either the ERα antagonist ICI 182, 780 or the selective estrogen receptor modulator (SERM) tamoxifen. Whether CLC-3 channels are involved in the tumorigenic action of 17-β estradiol on breast cells still needs investigation.

### Ion Channel Regulation by Androgens

Androgens play a fundamental role in cell growth and survival in androgen-receptor positive (AR+) prostate cancer cell lines, early-stages of prostate cancer, and even in normal prostate. However, aggressive and late-stage prostate cancers become androgen-independent (Feldman and Feldman, 2001; Yee, 2015). Interestingly, the changes in the sensitivity to androgens parallel modifications in the ion channel expression profile of prostate cancer cells. Different studies showed that androgens exert a transcriptional control on the expression of the TRPM8 calcium channel. Interestingly, the trpm8 gene has 10 putative androgen-responsive elements, one located in the promoter region, and the remaining in introns. Single cell RT-PCR and immunohistochemical analysis revealed that TRPM8 is expressed in androgen-sensitive human prostate apical secretory epithelial cancer cells, but its expression is decreased in cells that lost the androgen receptor. Moreover, TRPM8 channels are down-regulated as prostate cancer cells turn into androgen-independent cells (Gkika and Prevarskaya, 2009). In agreement with these results, Asutkhar et al. (2015) showed that testosterone (1 pM) enhanced Ca^+2^ uptake through endogenous TRPM8 in primary human prostate cells. The effect of testosterone was inhibited by pre-incubating cells with the TRPM8 blocker N-(2-aminoethyl)-N-(4-(benzyloxy)-3-methoxybenzyl)thiophene-2-carboxamide hydrochloride, M8-B (1 μM). Interestingly, these authors suggested that the TRPM8 channel might act as a testosterone receptor, since immunoprecipation with anti-DHT/testosterone IgG showed high levels of TRPM8 protein from different cells including the prostate cancer cell lines LNCaP and PC3, the prostate epithelial cell line RWPE-2 and HEK cells stably expressing TRPM8 channels, after being incubated with testosterone (1 μM) for 3 h. ELISA experiments using purified TRPM8 protein revealed a direct interaction of testosterone with TRPM8. The binding of testosterone was decreased by the presence of TRPM 8 agonists such as menthol (50 μM), icilin (10 μM), and M8-B (1 μM). However, Grolez et al. (2019) showed that, testosterone (10 nM) inhibited the transient increase of the intracellular calcium concentration induced by icillin (10 μM) in the presence of the AR. These results suggest that at some concentrations, testosterone inhibits the activity of TRPM8 *via* an AR-dependent mechanism. Moreover, testosterone (10 μM) facilitates the migration of prostate cancer cells by inhibiting TRPM8 channels. This inhibition was produced by the activated AR interacting with the channel present within lipid-raft domains. Grolez et al. (2019) proposed that the differences between their results and those from Asutkhar et al. (2015) might be explained by the differences in the design of the experiments since the former study used lipid bilayers and cells lacking the AR receptor, as well as lower concentrations of testosterone. Thus, the elucidation of the precise molecular mechanism of androgen regulation of TRPM8 channels requires further investigation.

TRPV6 channels are thought to be involved in cell proliferation under physiological and pathological conditions. Schwartz et al. (2006) showed that TRPV6 channels contribute to the increased proliferation rate of HEK-293 cells in a calcium-dependent manner. In prostate cancer cells, TRPV6 channels are also associated with increased cell proliferation, and resistance to apoptotic stimuli (Lehenkyi et al., 2007; Stewart, 2020). Interestingly, in some cases, the expression of TRPV6 seems to be regulated in a ligand-independent manner because the AR-selective agonist dihydrotestosterone (1 nM), or the AR selective antagonist casodex (10 μM, 24 h), showed no significant effects on TRPV6 expression (Lehenkyi et al., 2007) In contrast, in another study, dihydrotestosterone was shown to inhibit the expression of TRPV6, whereas the AR antagonist bicalutamide promoted its expression (Lehenkyi et al., 2012). In accordance with the latter, the expression of TRPV6 channels is significantly high in most androgen-independent lesions (Maly and Hofmann, 2018). Therefore, the molecular mechanism underlying the effect of androgens on TRPV6 channel expression needs to be elucidated. In addition, electrophysiological data are required to elucidate TRPV6 channel activity in malignant tissues, and how its activity is coupled to cancer progression. Androgens also regulate the expression of the voltage-gated calcium channels Cav3.2. In the prostate cancer cell line LNCaP, neuroendocrine differentiation (or trans-differentiation) induced either, by androgen-free serum or cAMP, was accompanied by an increased proportion of cells expressing Cav3.2 channels, characterized by patch-clamp recordings, pharmacological blockers, and small-interfering RNA (Buchanan and McCloskey, 2016). Trans-differentiation is a contributing factor to the transition of prostate cancer to the androgen- independent phenotype. At this stage, neuroendocrine cells release mitogenic factors, which may lead to cancer progression and poor prognosis. Cav3.2 channels may have an important role in this process by allowing calcium entry, and favoring mitogen release (Antal and Martin-Caraballo, 2019). Dihydrotestosterone (1 nM for 24 to 72 h) also up-regulates the expression of the Cav1.2 (α1C) subunit of the L-type calcium channels in MCF-7 cells. Further investigation is required to assess the impact of this channel in the calcium signaling occurring in breast cancer cells (Marques et al., 2015).

### Regulation of Ion Channels by Vitamin D and Calcitriol

Vitamin D and its active metabolite calcitriol regulate the expression of TRPV6 channels in cancer cells. The human colon cancer cell line CaCo-2 treated with calcitriol (10^-7^ M) for 8 h showed an extraordinary 60-fold increase of TRPV6 mRNA levels, an effect that was completely inhibited by actinomycin (Taparia et al., 2006). A similar result was observed also in CaCo-2 cells overexpressing the VDR and treated with the VDR-agonist curcumin (5 X 10^-5^ M) (Bartik et al., 2010). Interestingly, calcitriol and curcumin have anti-proliferative effects on colon cancer cells. Thus, it is intriguing how TRPV6 channels (which contribute to cell proliferation) may participate in the anti-proliferative effect of vitamin D. Besides, in a low-steroid environment, calcitriol promotes cell proliferation in LNCaP cells, by increasing the expression of TRPV6 channels, which augments calcium uptake (Lehenkyi et al., 2011). It is worth mentioning that calcitriol (100 nM) also up-regulates the expression of TRPV6 in T47D breast cancer cells (Bolanz et al., 2008). Recently, Chen et al. (2018) showed that the expression of the calcium channel TRPV5 inversely correlates with the expression of the VDR in renal cancer cells. Knockdown of TRPV5 in these cells inhibited cell proliferation, migration, and invasion induced by VDR knockdown. These results suggest that VDR acts as a tumor suppressor in renal cancer cells, and that this action includes the suppression of TRPV5 channel expression. Therefore, vitamin D regulates calcium influx by modulating the expression of TRPV6 and TRPV5 channels in a tissue-dependent manner. Calcitriol (10^-7^ M, 24 h) also down-regulates the expression of the Kv10.1 channel in breast and cervical cancer cells (Avila et al., 2010; García-Becerra et al., 2010). Since the VDR antagonist ZK 159222 (10^-5^ M) abrogated the effect of calcitriol on Kv10.1 expression, the authors suggested that this event may occur at transcriptional level. Remarkably, the reduced expression of Kv10.1 induced by calcitriol, parallels the decrease of cell proliferation in cervical cancer cells. Thus, it is possible that the anti-proliferative effect of vitamin D on cervical cancer may be associated to the down-regulation of Kv10.1 channels (Avila et al., 2010; García-Becerra et al., 2010).

**Figure 1:**
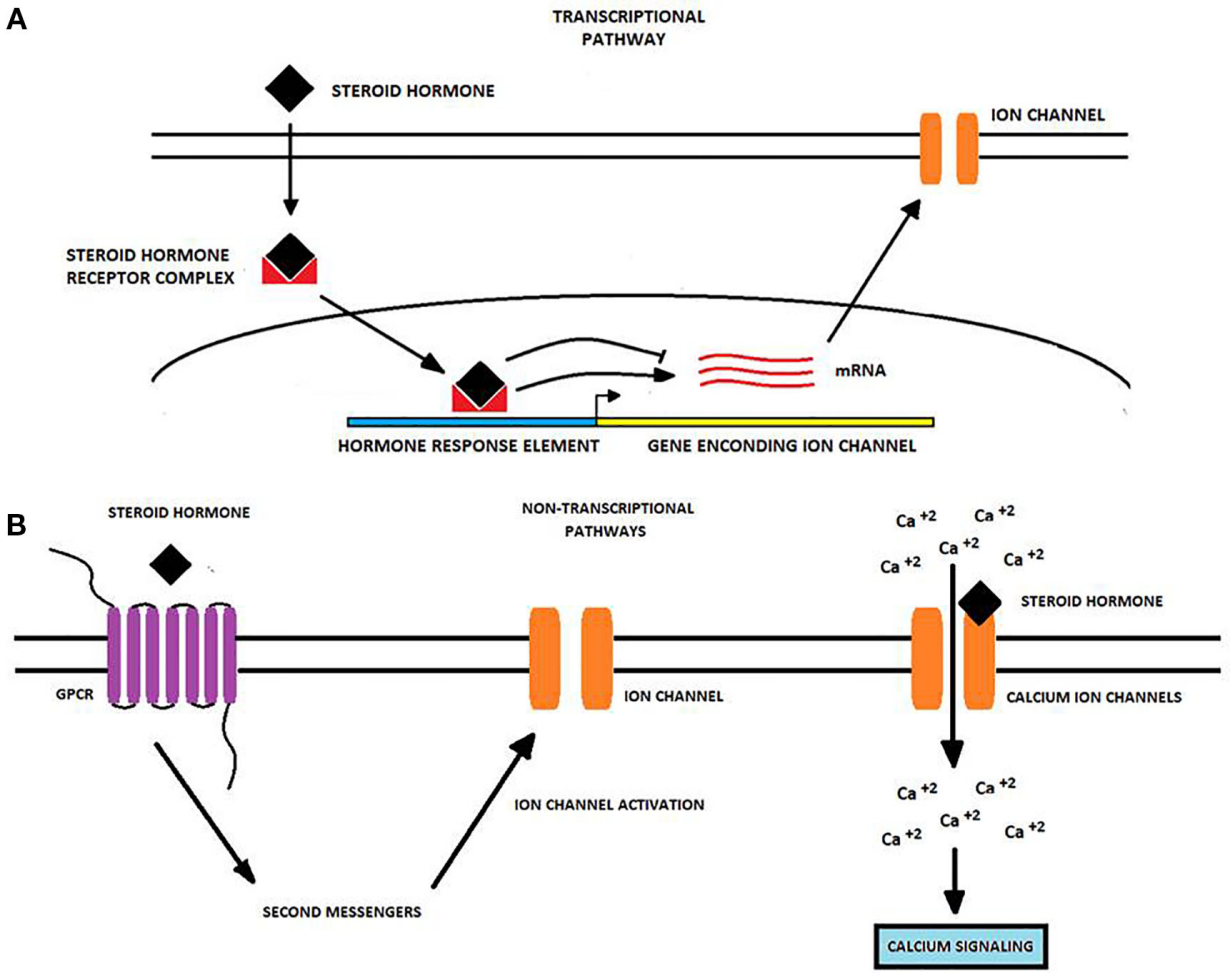
Transcriptional and non-transcriptional regulation of ion channels by steroid hormones in cancer cells. Steroid hormones (SH) regulate the expression and activity of many ion channels *via* two potential molecular pathways. **(A)** The *transcriptional pathway* starts when an SH passively diffuses into the cell and binds an intracellular receptor forming an SH-receptor complex that moves into the nucleus. Here, the complex binds to hormone-response elements to either induce or repress the transcription of genes encoding for ion channels. Upon transcription, ion channel mRNA is translated into the corresponding protein and the ion channel is transported to the cell membrane (Bohra and Bhateja, 2015; Wilkenfeld et al., 2018). **(B)** *Non-transcriptional pathways* primarily involve the binding of the SH to a GPCR triggering second messenger pathways which may activate proteins targeting to ion channels. Alternatively, SH may bind to ion channels like TRPM8 and CatSper modulating its gating properties and enhancing calcium influx (Head et al., 1999; Asuthkar et al., 2015; Grolez et al., 2019). Changes in ion channel expression or activity produced by SH may in turn affect some cancer-associated processes, including cancer cell proliferation, migration, and invasion.

**Table 1** summarizes data from a number of studies concerning the regulation of different ion channels by SSH and calcitriol in different types of cancer, while **Figure 1** depicts the diverse mechanisms by which SH may regulate ion channel expression and/or activity.

**Table 1:** Sex steroid hormone and calcitriol regulation of ion channels in cancer cells.

Hormone Concentration/dose, time of exposure	Ion channel	Effect	Cell type	Cellular process	Reference
**17-β Estradiol**					
10-14 – 10-10 M, 48 h	Kv10.1	Upregulation	HeLa transfected with ERS1 gene	N/D	Díaz et al., 2009
0.05 mg, 60 days (pellet)		Upregulation	Cervical tissue of HPV-E7 transgenic mice	Tumor progression	Ramírez et al., 2018
Not determined in the patients		Upregulation	Cervical pap-smears from patients taking estrogens	N/D	Ortiz et al., 2011
0.05 mg, 60 days (pellet)	KCa1.1	Upregulation	Cervical tissue of HPV-E7 transgenic mice	Tumor progression	Ramírez et al., 2018
1 nM, 48 and 72 h	KCa1.1	Upregulation	Endometrial cancer Ishikawa cells	Proliferation, migration and invasion	Wang et al., 2018
10 nM, extracelullar perfusion		Increased currents	MCF-7	N/D	Coiret et al., 2005
100 nM,overnight	Orai3	Upregulation	MCF-7	Calcium influx and anchorage-independent growth	Motiani et al., 2013
10 nM, 24 and 48 h	TRPM8	Induced expression	MCF-7		Chodon et al., 2010
100 nM, (time- dependent)	Cav 1.3	Upregulation	Endometrial cancer cells	Calcium influx, proliferation and migration	Hao et al., 2015
10 nM, extracellular perfusion, 10 s	Nav 1.5	Increased currents via GPR30	MDA-MB-231	Decreased cell adhesion	Fraser et al., 2010
100 nM, gradually activated from 500 – 2000 s	CLC-3	Upregulation	MDA-MB-231 transfected with ERS1 gene	N/D	Yang et al., 2018
**Dihydro-testosterone**					
1 nM, 24 h to 72 h	Cav 1.2	Upregulation	MCF-7 cells	N/D	Marques et al., 2015
1 μM, 3 h	TRPM8	Upregulation	LNCaP and PC3	N/D	Asuthkar et al., 2015
10 nM, 15 min	TRPM8	Inhibition of Ca+2 currents	PC3		Grolez et al., 2019
**Calcitriol**					
10-7 M, 8 h	TRPV6	Upregulation	CaCo-2	N/D	Taparia et al., 2006; Bartik et al., 2010
100 nM, 2 - 4 days		Upregulation	LNCaP	Cell proliferation	Lehenkyi et al., 2011
100 nM, 24 h		Upregulation	T47D	N/D	Bolanz et al., 2008
10-7 M, 24 h	Kv10.1	Downregulation	SiHa cells transfected with VDR	Decreased cell proliferation	Ávila et al., 2010
		Downregulation	Breast tumor derived cells	Decreased cell proliferation	García-Becerra et al., 2010

N/D, Not determined.

## Steroid Hormone Regulation of Ion Channels As A Potential Opportunity For Early Tumor Diagnosis, Prognosis and Cancer Therapy

### Ion Channels in Early Detection and Prognosis of Steroid-Responsive Cancers

Since SSH induce carcinogenesis in some tissue, it is expected that some genes regulated by these hormones are either expressed or repressed at the initial stages of the disease, gaining value as potential early cancer biomarkers. In accordance with the increased expression of Kv10.1 channels from low-grade dysplasia to cervical cancer in transgenic mice treated with estrogens (0.05 mg pellets subcutaneously inserted) (Ramírez et al., 2018), Kv10.1 protein expression is also increased in human samples from cervical dysplasia and cancer, as well as in patients taking estrogens. Kv10.1 protein expression was detected in 67% of human cervical cytologies from low-grade intraepithelial lesions, and in 92% of samples from high-grade intraepithelial lesions, but only in 27% of normal non-cancerous samples (Ortiz et al., 2011). In tissue biopsies from different grades of cervical intraepithelial neoplasia (CIN 1-3), it was found that the higher the grade of neoplasia, the higher the expression of Kv10.1 protein (Ortiz et al., 2011). In the same work, it was reported that almost 50% of the patients with normal cervical cytologies, but taking estrogens as either, hormone-replacement or contraceptive therapy, were positive for Kv10.1 protein expression, versus 27% of non-users. These data yielded a statistically significant association between the use of estrogens and Kv10.1 expression. In addition, the presence of the Kv10.1 transcript has been reported in the “tumor free” mammary gland surrounding the breast carcinoma tissue (Hemmerlein et al., 2006). Thus, this channel has been also proposed as an early marker for breast cancer. Besides, Kv10.1 immuno-intensity increases with the grade of breast cancer and results higher in triple-negative samples which belong to the most aggressive phenotype (Liu et al., 2015; Li et al., 2017); however, the potential association between Kv10.1 and ERα expression in breast cancer needs further investigation. Likewise, KCa 1. 1 channels have also been proposed as potential early cervical cancer markers (Ramírez et al., 2018). Protein expression has not been found in human non-cancerous cervix. In contrast, channel protein expression gradually increased from low-grade to high-grade dysplasia and to carcinoma tissues.

Prostate serum antigen (PSA) has been the gold standard technique for detecting prostate cancer in a non-invasive manner; however, new markers that increase the rate of true positive results are still required. TRPM8 channels are promising prostate cancer biomarkers. Bai et al. (2010) found that the mRNA of TRPM 8 is up-regulated in prostate tumor tissue compared to patient-matched non-tumor tissue, however, it did not correlate with the Gleason score. Interestingly, TRPM8 mRNA can be detected in urine and blood of patients with metastatic disease. Probably, the mRNA of TRPM8 is packaged into the extracellular vesicles released by prostate cancer cells, but at the moment this possibility has not been addressed (Kalra et al., 2012). TRPV6 channels have been proposed as prognostic biomarkers for prostate cancer, as well. Fixemer et al. (2003) found that TRPV6 expression significantly correlated with the Gleason score, pathological stage and extra prostatic extension in prostatectomy specimens from 97 clinically organ-confined tumors. Likewise, lymph node metastasis and androgen-insensitive tumors revealed TRPV6 expression in 63 and 67% of cases, respectively. Moreover, Raphaël et al. (2014) demonstrated that TRPV6 channels may be slightly expressed in benign prostate hyperplasia, but its expression increases in prostate carcinomas. These authors concluded that the translocation of the channel to the plasma membrane *via* the Orai1-mediated Ca^2+^/Annexin I/S100A pathway is the molecular mechanism responsible for the oncogenic potential of TRPV6 channels in prostate cancer. All these results suggest TRPM8 and TRPV6 channels as potential markers for prostate cancer progression and prognosis.

The expression of KCa 1.1 channels was studied in 263 endometrial tissue samples, including 185 type I endometrial cancer, 40 normal endometrial tissues, and 38 atypical hyperplasia samples. KCa1.1 protein expression was significantly elevated in endometrial adenocarcinoma, compared to normal tissue and atypical endometrial hyperplasia. No differences were found between normal endometrial tissue and atypical endometrial hyperplasia. As expected, phosphorylation of ERK and MEK were also increased in endometrial adenocarcinoma samples, compared to normal and hyperplastic endometrial tissue. Interestingly, increased expression of the KCa 1.1 channel was significantly associated with FIGO stage ≥ II and lymph node metastasis (LNM). Additionally, p-ERK up-regulated expression was significantly associated with FIGO stage ≥ II, cervical stromal involvement, lymphovascular space invasion (LVSI), and LNM. These results suggest that up-regulated expression of KCa1.1 channels and p-ERK is related with poor prognosis in type I endometrial cancer (Wang et al., 2018). In summary, ion channel detection in hormone-sensitive tissues or extracellular fluids may serve as potential early markers of SSH-associated cancers. Finally, the regulation of ion channels by SSH in other hormone-sensitive tissues like adrenal gland and testis requires further investigation.

### Vitamin D Anti-Proliferative Effects and Ion Channels: A Promising Therapeutic Option for Steroid-Responsive Cancers

Many studies show that calcitriol is a promising co-adjuvant for the treatment of tumors expressing VDR; nevertheless, the use of vitamin D as an adjuvant in cancer therapy is a matter of discussion (Young and Xiong, 2018). The arguments of this discussion are diverse. On one hand, calcitriol has pro-proliferative effects [for instance in prostate cancer cells as above mentioned (Lehenkyi et al., 2011)], and induces hypercalcemia increasing the risk of cardiovascular, gastrointestinal, renal, and neurological- side-effects (Feldenzer and Sarno, 2018). On the other hand, vitamin D has anti-proliferative effects, prevents tumor lesion progression, and improve the quality of cancer patients (Young and Xiong, 2018), although the results are variable between clinical trials. Calcitriol acts as an anti-cancer agent by modulating important regulatory networks that result in the inhibition of cell proliferation, apoptosis and cell differentiation. The transcriptional regulation of some ion channels seems to be a mechanism by which calcitriol has anti-tumoral effects. Garcia-Quiroz et al. (2012) reported that astemizole (an anti-histamine that blocks some members of the EAG channel family) synergistically interacts with calcitriol, at clinically achievable drug concentration, to inhibit cell proliferation of the human breast cancer cells lines T-47D and SUM-229PE. Furthermore, astemizole signicantly increased the growth inhibitory effect of calcitriol by three-fold with a mean inhibitory concentration 20 (IC_20_) of 1.62 ± 0.75 μM and 1.82 ± 2.41 nM, respectively. When concomitantly used, astemizole (IC_50_ = 2.02 μM) and calcitriol (1 and 10 μM) down-regulate the expression of Ki-67 (a cell proliferation marker) and Kv10.1. Interestingly, astemizole (3 μM) up-regulated the expression of VDR, which might explain the synergic effect. The combined anti-tumoral effect of astemizole and 1,25 VDR was studied in mice xenografted with TD-47 cells and a primary breast cancer derived-cell culture (MBCDF). Tumor-bearing athymic female mice were treated with oral astemizole (50 mg/kg day) and calcitriol peritoneal injections (0.03 µg/g body weight twice a week) for three weeks. Compared to untreated controls, combined therapy significantly reduced tumor growth. As for cell lines, reduced tumor growth also was accompanied by decreased expression levels of Kv10.1 channels and Ki-67 (García-Quiroz et al., 2014). The synergistic anti-proliferative effect of astemizole and calcitriol has been also observed in hepatocellular carcinoma (Xu et al., 2018), however, the potential involvement of ion channels in this synergy in liver cancer remains elusive.

Calcitriol may also be a useful molecule for the treatment of prostate cancer. Mouse models of full penetrant and slowly evolving prostate tumorigenesis showed that high-calcium diet accelerates the progression of prostate intraepithelial neoplasia by promoting cell proliferation, micro-invasion, tissue inflammation, and the expression of prostate cancer markers. Remarkably, vitamin D prevented the high-calcium diet tumorigenic effect. Data strongly suggested that the antitumor effect of vitamin D and calcitriol included a transcriptional down-regulation of TRPC6 calcium channels. Moreover, the proliferation of PC-3 prostate cancer cells was decreased by silencing the expression of TRPC6 (Bernichtein et al., 2017).

Khatun et al. (2016) showed that calcitriol (1 uM, 72 h) and calcipotriol (1 uM, 72 h), a VDR agonist, inhibited the expression of KCa1.1 channels in MDA-MB-453 breast cancer cells. Then, it was suggested that the transcriptional repression and protein down-regulation of KCa1.1 channels partly contributes to the anti-proliferative effect of the VDR agonist on breast cancer cells.

## Concluding Remarks

A plethora of evidences from cancer cell lines and animal models, as well as human tumor samples, strongly suggests that ion channels are essential components for the association of SSH with cancer. Further translational and clinical research is needed to assess the impact that ion channels blockers, combined with calcitriol or anti-hormone therapy, may have in the treatment of cancer. Nevertheless, by considering that SSH are risk factors for different types of cancer, and ion channels play important roles in tumor progression, the regulation of ion channels by these hormones and calcitriol may serve as an opportunity for early cancer diagnosis and therapeutic approaches in SH-sensitive tumors.

## Author Contributions

IR-A, CB, and JC contributed conception and design of the review. IR-A wrote the first draft of the manuscript. CB and JC wrote sections of the manuscript. All authors contributed to manuscript revision, read and approved the submitted version.

## Conflict of Interest

The authors declare that the research was conducted in the absence of any commercial or financial relationships that could be construed as a potential conflict of interest.

## Abbreviations

1,25 VD, 1α,25 dihydroxivitamin D; AR, Androgen receptor; Cav, Voltage-gated calcium channel; ERα, Estrogen receptor alpha; ERβ, Estrogen receptor beta; ERK, Extracellular Receptor Kinase; FAK, Focal Adhesion Kinase; GPR30: G protein-coupled receptor 30; HPV, Human papilloma virus; Kca, Calcium-activated potassium channel; Kv, Voltage-gated potassium channel; MAPK, Mitogen Activated Protein Kinase; Nav, Voltage-gated sodium channel; PKA, Protein Kinase A; SH, Steroid hormones; SSH, Sex steroid hormones; TRP, Transient receptor potential; VDR, Vitamin D receptor.
